# Bayesian Inference for Genomic Data Integration Reduces Misclassification Rate in Predicting Protein-Protein Interactions

**DOI:** 10.1371/journal.pcbi.1002110

**Published:** 2011-07-28

**Authors:** Chuanhua Xing, David B. Dunson

**Affiliations:** 1 Department of Biostatistics and Bioinformatics, Duke University, Durham, North Carolina, United States of America; 2 Department of Statistical Science, Duke University, Durham, North Carolina, United States of America; The Centre for Research and Technology, Hellas, Greece

## Abstract

Protein-protein interactions (PPIs) are essential to most fundamental cellular processes. There has been increasing interest in reconstructing PPIs networks. However, several critical difficulties exist in obtaining reliable predictions. Noticeably, false positive rates can be as high as >80%. Error correction from each generating source can be both time-consuming and inefficient due to the difficulty of covering the errors from multiple levels of data processing procedures within a single test. We propose a novel Bayesian integration method, deemed nonparametric Bayes ensemble learning (NBEL), to lower the misclassification rate (both false positives and negatives) through automatically up-weighting data sources that are most informative, while down-weighting less informative and biased sources. Extensive studies indicate that NBEL is significantly more robust than the classic naïve Bayes to unreliable, error-prone and contaminated data. On a large human data set our NBEL approach predicts many more PPIs than naïve Bayes. This suggests that previous studies may have large numbers of not only false positives but also false negatives. The validation on two human PPIs datasets having high quality supports our observations. Our experiments demonstrate that it is feasible to predict high-throughput PPIs computationally with substantially reduced false positives and false negatives. The ability of predicting large numbers of PPIs both reliably and automatically may inspire people to use computational approaches to correct data errors in general, and may speed up PPIs prediction with high quality. Such a reliable prediction may provide a solid platform to other studies such as protein functions prediction and roles of PPIs in disease susceptibility.

## Author Summary

Protein interactions are the basic units in almost all biological processes. It is thus vitally important to reconstruct protein-protein interactions (PPIs) before we can fully understand biological processes. However, critical difficulties exist. Particularly the rate of wrongly predicting PPIs to be true (false positive rate) is extremely high in PPIs prediction. The traditional approaches of error correction from each generating source can be both time-consuming and inefficient. We propose a method that can substantially reduce false positive rates by emphasizing information from more reliable data sources, and de-emphasizing less reliable sources. We indicate that it is indeed the case from our extensive studies. Our predictions also suggest that large numbers of not only false positives but also false negatives may exist in previous studies, as validated by two human PPIs datasets having high quality. The ability to predict large numbers of PPIs both reliably and automatically may inspire people to use computational approaches to correct data errors in general, and speed up PPIs prediction with high quality. Reliable prediction from our method may benefit other studies involving such as protein function prediction and roles of PPIs in disease susceptibility.

## Introduction

Protein interactions play important roles in most fundamental cellular processes. There has been increasing interest in reconstructing the interactome of a cell as large-scale data become available [1]. The improved knowledge of protein-protein interactions (PPIs) assists in detecting the susceptibility to human complex diseases [2]–[3] and then in discovery of new drugs and pharmaceuticals [4]–[8]. A variety of high-throughput experimental approaches have been developed to identify sets of interacting proteins, including yeast two-hybrid (Y2H) screening and mass spectrometry methods. However, these approaches are known to suffer from high false positives [9]–[12] and also high false negatives. A wide variety of computational approaches have been proposed for predicting PPIs. Some are based on data mining from published literature [13]–[17]. Please refer to [18] for a more complete review. The other studies are based on the amino acid sequences combined with additional information, such as co-expression patterns, phylogenetic distributions of orthologous groups, co-evolution patterns, the order of genes in the genome, gene fusion and fission events, and synthetic lethality of gene knockouts [19]–[27]. For reviews, refer to Bork et al. 2004, Shoemaker and Panchenko 2007, and Valencia and Pazos 2002 [28]–[30]. In this article, we focus on integrating the information from disparate data sources for the prediction of protein-protein interactions.

Genomic data integration has become popular in recent years with the intention of improving the power in predicting PPIs, as more disparate PPIs data are available. Several such methods have been recently developed, including decision trees [31]–[33], support vector machines (SVMs) [34], Bayesian models [22], [35]–[38], [39]–[44] and other considerations such as improving gold standard negative (GSN) set [45]. Among them, Bayes models have provided the most widely used paradigm for probabilistically integrating diverse data types. To calculate the score for each protein pair in each data source, the protein pairs are typically divided into subgroups based on features. One then calculates the likelihood ratio for the protein pairs in each feature subset by evaluating the ratio of the proportion of protein pairs in gold positive data set and the proportion in gold negative data set. Gold positive (negative) set is a dataset that includes protein pairs that are highly believed to be interacting (non-interacting). Naïve Bayes then multiplies directly the scores from multiple data sources for predicting whether a protein pair is interacting or not. A protein pair is defined as interacting by observing a >1 posterior odds ratio, after multiplying prior odds to the likelihood ratio. Lee et al. 2004 and Lu et al. 2005 [36]–[37] integrated diverse functional genomics to reconstruct a functional gene network for Saccharomyces cerevisiae. Their results are comparable in accuracy to small-scale interaction assays with an increased true positive rate. Rhodes et al. 2005 [38] employed a naïve Bayes model to combine four data sources, ortholog interactions, co-expression, shared biological function, and enriched domain pairs. With a careful selection of prior information, their naïve Bayes model predicts nearly 40,000 protein-protein interactions in humans. They reported a false positive rate of 50%, though Hart et al. 2006 [39] later estimates this rate to be 85%. Also using a naïve Bayes approach, Scott and Barton, 2007 [40] predicted 37,606 human PPIs, with an estimated false positive rate as high as 76%.

As reviewed above, the predictions of PPIs still suffer a rather high false positive rate, which can be as high as >80%. In addition, current PPIs prediction is far from complete with yeast ∼50% and human only ∼10% identified [1], [39]. Hence, it is of critical importance to effectively reduce the false positive rate for a more reliable and complete prediction of large numbers of PPIs. Some of the errors may result from inaccurate data collection and error-prone data sources, though it is reasonable to assume that such data are in the minority and the majority properly reflects the evidence of interactions. To reduce the misclassification rate, it is necessary for the method to be robust to biased and non-informative data sources. The popular naïve Bayes model flexibly integrates the interaction information in a probabilistic way, compared with other data integration methods. However, the direct multiplication of likelihood ratio scores may not be able to effectively handle the effects of errors including missing interactions, sampling biases, and false positives [1]. Such errors can lead to completely wrong predictions even if they may come only from one single data source. It is therefore critically important to develop a novel algorithm that is able to effectively minimize the effects of the errors in data and therefore reduce the misclassification rate for obtaining a reliable prediction of PPIs.

We propose a nonparametric Bayes latent class discriminant analysis approach, which we refer to as nonparametric Bayes ensemble learning (NBEL) due to the ability to flexibly ensemble information about the presence of PPIs across different data sources. The goal of NBEL is to lower the false positive rate through automatically up-weighting the data sources that are most informative about a PPI, while down-weighting less informative and biased sources. None of existing integration methods, as far as we know, is able to flexibly weight the data sources for optimally capturing the information of PPIs. Bader et al. 2004 [46] weighted their positive and negative training examples inversely according to their fraction of the training set to favor 0.5 as the prior dividing threshold. InPrePPI [47] used a naïve Bayesian fashion to integrate multiple data sources by multiplying a weight, which is approximately estimated for each data source. However, the contributions of data sources can be different for the different protein pairs due to their different biological functions. NBEL learns the distributions of the likelihood ratios (LRs) for interacting and non-interacting protein pairs within each data source. If the distribution of the LRs for interacting and non-interacting pairs is not well separated for a particular data source, then that source will be down-weighted automatically in calculating the posterior probability of a PPI. In this manner, NBEL does not equally weight the different data sources, but instead learns the weights adaptively in a probabilistic manner. NBEL is thus more robust than classic naïve Bayes to unreliable, error-prone and contaminated data, and our extensive studies indicate this is indeed the case. On a large human data set our NBEL approach predicts many more PPIs than naïve Bayes, which suggests that large numbers of not only false positives but also false negatives may exist in previous studies. The validation on two experimental datasets having high quality supports our observation.

## Results

We conducted extensive simulation studies to evaluate and validate the performance of the proposed NBEL method. We compared the results with two methods, naïve Bayes and logistic regression. We then tested our approach on human genomic data sets. We finally validated the performance of NBEL via two experimental human PPIs data having high quality.

### Simulation Studies

The goal of our simulations is to assess the performance of our NBEL algorithm compared with two popular methods, naïve Bayes and logistic regression, in cases in which the interaction status is known. Current genomic integration approaches usually evaluate their prediction by comparing with the protein pairs in gold positive and negative datasets. However, only using gold positive and negative datasets may be misleading, as such data sets do not represent random samples of the entire set of human PPIs. In addition, the standards of selecting interacting protein pairs from each data source are rather ad hoc, and there is no known interacting information available for evaluation. One can verify the prediction using a small portion of experimental PPIs, but it is obviously not enough for evaluating large amount of computationally predicted PPIs. Hence, we also extensively tested on simulation data in which the truth is known.

We set up the simulations with 4 data sources. For the types of methods we are proposing, the performance of NBEL should improve as more data sources become available. We consider 5000 total protein pairs. We set the status of the first 1250 protein pairs as interacting, with the remaining 3750 non-interacting. We generated the simulated data using the model expressed by Equation (2) in the Methods section (The parameters used are summarized in Table 1 in Text S1). We chose the distributions to allow a varying degree of separation in the interacting and non-interacting distributions for the different data sources. As discussed in detail in the Methods section, the more separated the distributions are, the more informative the data source is about a PPI. With a high degree of separation in which the two component distributions have minimal overlap, misclassification errors will be low for any reasonable method, so our focus is on the more realistic case in which there is substantial overlap.

#### Tests on uncontaminated data

We applied our NBEL approach and compared the performance with the naïve Bayes and logistic regression under different simulation scenarios, with the first case assuming uncontaminated data. Uncontaminated data refers to the data that are simulated error free. We calculated the estimated posterior probability for an interacting protein pair by averaging its conditional probabilities over MCMC iterations after burn-in. We then predicted that there is an interaction in protein pair *i* if the estimated posterior probability is above a threshold. As noted in the Methods section, a 0–1 loss function results in an optimal threshold of 0.5, with this choice minimizing the Bayes risk defined as the posterior expectation of the overall misclassification rate obtained by weighting false positives and negatives equally.

**Figure 1:**
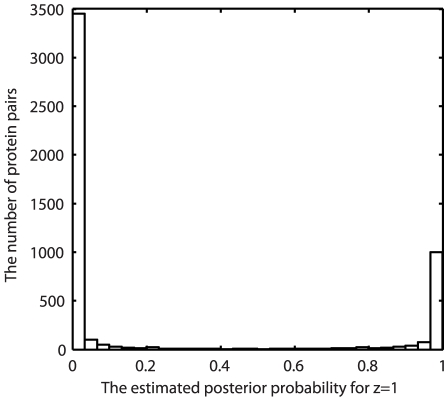
The histogram of the estimated posterior probabilities of interacting protein pairs from NBEL algorithm. This is from an example simulation using our NBEL. We can observe a clear bimodal pattern with almost all of the interacting pairs having posterior probabilities close to one and almost all of the non-interacting pairs having posterior probabilities close to zero.

The histogram of the estimated posterior probabilities for an example simulation is shown in Figure 1. There is a clear bimodal distribution with most of the interacting pairs having values close to one and most of the non-interacting pairs having values close to zero. The optimal 0.5 threshold separates them well. To compare with naïve Bayes, we directly multiplied the likelihood ratios (LRs) from the different data sources to obtain a final score for a protein pair. We then estimate a threshold that maximally separates the two modes in the histogram of all the final scores (we call this estimated threshold as *the alternative threshold* in short later on in this paper). To assess the impact of threshold choice on the performance and build a direct connection for comparing with naïve Bayes, we also evaluated the performance using the alternative threshold in applying our NBEL method. We chose 0.5 as the threshold for logistic regression, which typically produces very close results to using *the alternative threshold* based on our observations.

We analyzed 50 simulated data sets using three methods. Our NBEL method had lower misclassification rates (misclassification rate is defined as the average of the false positive (FP) rate and the false negative rate (FN)) than both naïve Bayes and logistic regression in all 50 simulated data sets, with the averaged misclassification rates 1.99% for NBEL using the threshold 0.5, 2.25% for NBEL using the alternative threshold, 7.57% using the alternative threshold for naïve Bayes, and 5.85% for logistic regression using the threshold 0.5. We can observe that the NBEL misclassification rates using the two thresholds are very close, and both are much better than naïve Bayes with an average difference of 5.58% and logistic regression with an average difference of 3.86%. Given the ideal case that the data sources are uncontaminated, such misclassification rate reduction from 7.57% or 5.85% to 1.99% can be a remarkable improvement especially when there is thousands and millions of PPIs data in the real data tests.

#### Tests on contaminated data

PPIs data in the real world however involves a large portion of false positives with possible varying false positive rates as we discussed in the Introduction section, although we expect that the situation can be better as the research goes on. We therefore simulated a series of data involving varying levels of contaminated data to examine the performance of our NBEL in reducing misclassification. We carried on the tests by repeating the same procedure as above but inducing errors to the data, in which a randomly-selected proportion of the protein pairs had their interaction status reversed. We created five sets of contaminated data with different levels of errors, and tested the performances of our NBEL algorithm on them. For the first data set, we randomly picked 25 out of 1250 interacting protein pairs for each data source, and reversed their status into non-interacting. We then randomly picked 75 out of 3750 non-interacting protein pairs for each data source, and, similarly, reversed their status into interacting. The induced error rate is slightly greater than 7% over all data sources, with the majority of errors occurring in fewer than two out of four data sources. The error rate is measured as the average of the induced false positive and false negative rates. We generated the remaining contaminated data sets by multiplying the number of protein pairs with scores appropriately reversed by 2, 4, 8, and 16 times of that for the first contaminated data set. Data were otherwise simulated and analyzed exactly as in *Uncontaminated Data*. The generated data are summarized in Table 1.

**Table 1:** Summary of the contaminated data sets.

	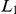 (out of 1250)	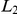 (out of 3750)	Induced Error Rate
Contaminated Data I	96	288	7.68%
Contaminated Data II	182	551	14.63%
Contaminated Data III	342	1031	27.43%
Contaminated Data IV	582	1775	46.95%
Contaminated Data V	894	2670	71.36%

In table 1, 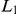 represents the number of interacting protein pairs that are reversed, and 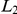 represents the number of non-interacting protein pairs that are reversed. We set the status for 1250 out of 5000 protein pairs as interacting, and 3750 out of 5000 protein pairs as non-interacting.

**Figure 2:**
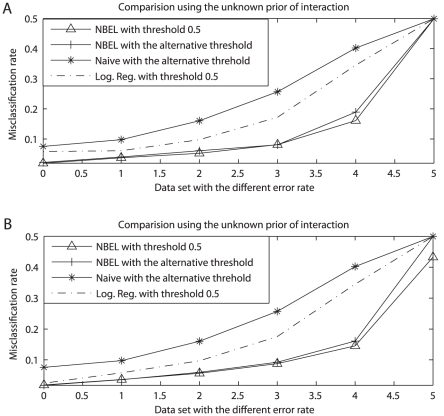
Comparison with Naïve Bayes and logistic regression when the data sets have the different induced error rates. The data set at x axis 0 represents noncontaminated data, and the data sets from x axis 1 to 5 represent contaminated data set I to V, with the induced error rates varying from 7.68% to 71.36%. y axis represents misclassification rate. A) is the comparison using the unknown prior of interaction. B) is the comparison using the known prior of interaction.

We applied three methods to each data set. This procedure was repeated on 50 independently generated data sets. The averaged misclassification rates, together with the ones for uncontaminated data, are summarized and plotted in Figure 2 (A). Similarly to uncontaminated data, our NBEL algorithm using either threshold has much lower misclassification rate than both naïve Bayes and logistic regression. Meanwhile, the misclassification rate reduction tends to be larger when the induced error rate in the data is higher, with a rather remarkable rate reduction of >22% from both naïve Bayes and logistic regression when the error rate in the data is as high as 46.95% in *Contaminated data IV*. The averaged standard deviation of FP and FN for 6 datasets varies from 0.0063 to 0.0158 for our NBEL, from 0.0078 to 0.0095 for logistic regression, and is high for naïve Bayes varying from 0.03 to 0.05. This suggests that our NBEL has a very strong function of error-correction, especially when the proportion of errors in the data is higher. This makes sense in that NBEL algorithm is designed to flexibly integrate multiple data sources by up-weighting the more informative but down-weighting the less informative and biased sources in calculating the posterior probability of a PPI. Such a weight adjustment procedure is carried through by examining how well the learnt distributions of interacting and non-interacting protein pairs are separated within each data source. NBEL is therefore able to minimize the effects of the problematic data source that may be the results of missing data, sampling bias, false positive, or simple data entry errors, while maximize the information from the authentic interacting PPIs. In contrast, both naïve Bayes and logistic regression are barely functional in correcting data errors, with logistic regression slightly better and the misclassification rates for naïve Bayes close to the given error rates in all data sets. This may explain why the false positive rate is so high in the previous PPIs predictions. NBEL algorithm therefore provides a more powerful tool to integrate multiple data sources for a better prediction of PPIs. This property can be critically important, especially when current data for PPIs prediction include heavy data errors.

However, when the data error rates are extremely high as illustrated as the last points in Figure 2(A), we can observe that the misclassification rate is close to non-informative random rate 50%. These overlapped points correspond to *Contaminated data V*, having the data error rate 71.36%. This random non-informative prediction can be expected because the ability to accurately detect PPIs intuitively requires the majority of the data sources to be informative with error rate less than 50%. However, performance can be improved to some extent in the presence of large amounts of contamination by eliciting prior information as to whether a protein pair interacts or not from the literature. To assess this, we repeated the above tests with a fixed prior. This prior pre-assigns a probability weight to a protein pairs as to how possible it can be interact or not. However, such exhaustive prior information is difficult or impossible to obtain for all protein pairs. We therefore simply pre-assign a weight that represents the weight of interacting or the proportion of interacting protein pairs in the whole dataset. We randomly choose such a prior that is close to the known proportion of 1/4 in this study. We then predicted the interacting protein pairs from the posterior analysis of the MCMC procedure. The results are summarized in Figure 2 (B).

We can observe from Figure 2 (B) that the performance is similar to the one using the unknown prior in the presence of lower contamination when the error rate is less than 50%. The standard deviations for 6 datasets also have the similar pattern to the tests using unknown prior but slightly smaller values. However, when the contamination is rather high as in *Contaminated data V*, the elicited prior leads to much better performance in having a noticeable reduction of ∼7% in misclassification rate. In addition to further supporting the previous conclusions, the elicited prior of being interacting or not may provide a realistic approach for genomic integration of PPIs data, especially when data includes rather high false positives and/or false negatives.

#### Tests as the number of data sources increases

**Figure 3:**
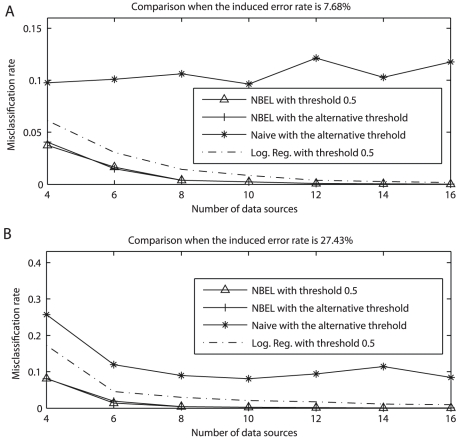
Comparison with Naïve Bayes and logistic regression when the number of data sources increases. X axis represents the number of data sources, and y axis represents misclassification rate. A) used contaminated data set I with the induced error rate 7.68%, and B) used contaminated data set III with the induced error rate 27.43%.

As the number of data sources increases, NBEL will have more evidence to predict whether a protein pair interacts or not. We varied the number of data sources to observe the influence on the misclassification rate. We used the first contaminated data, and apply NBEL and naïve Bayes when the number of data sources *p* is 4, 6, 8, 10, 12, 14, and 16, respectively. The simulation for every p is repeated 50 times. The averaged misclassification rates for the three approaches are plotted in Figure 3 (A). We can observe that the misclassification rates for our NBEL method using both the thresholds are much smaller than the ones for both naïve Bayes and logistic regression, with the misclassification rate for logistic regression obviously smaller than the one for naïve Bayes. As the number of data sources increases, the misclassification rates for NBEL and logistic regression reduce substantially. However, the misclassification rate for logistic regression is obviously higher than our NBEL, while naïve Bayes keeps a level of 8%∼10%. To observe whether the misclassification rate can be reduced to such a low rate when the contamination in data is high, we repeated the above test but using contaminated data set III, and the comparison of misclassification rates among three methods are plotted in Figure 3 (B). We can see that naïve Bayes has a certain level of error correction when the misclassification rate is rather high, as the number of data sources increases. However, it stops decreasing when it reaches a level of 8%∼10%, while NBEL and logistic regression decrease further. From Figure 3, logistic regression also produces a smaller misclassification rate as the number of data sources increases to be a large number such as 14 or greater. However, the number of reliable data sources to integrate in real data tests is usually not that large. While our NBEL is able to quickly reduce misclassification rate close to zero when the number of data sources increases to be 6 or 8. These tests supported our previous tests that our NBEL provides a more practical tool in predicting reliable PPIs from error-prone data, and learns from additional sources of informative data.

#### Receiver operating characteristic

**Figure 4:**
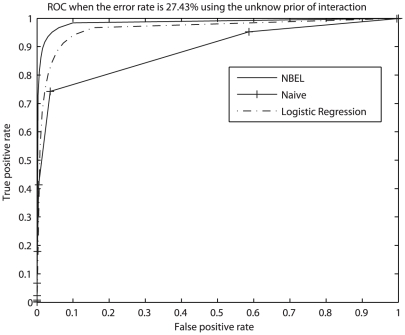
Receiver Operating Characteristic (ROC) curves for our NBEL algorithm, naïve Bayes, and logistic regression. We illustrate ROC curves using the data having error rate of 27.43% without the prior interaction information.

In this part, we show the performance for all methods using a receiver operating characteristic (ROC) curve, which is the plot of the true positive (TP) rate versus false positive (FP) rate. We observed the ROC curves for all 6 sets of data in Table 1, with and without the known prior information of interaction. We illustrate our observations of ROC curves using the contaminated data III in Figure 4, which has error rate equal to 27.43% with the unknown prior information of interaction. From Figure 4, we can observe that our NBEL has a better performance than logistic regression, and logistic regression has a better performance than naïve Bayes. This is consistent with the observations in the previous tests. When the error rate in a dataset is lower, the curves are closer to the left top corner; when the error rate is higher, the curves are closer to the diagonal line which is TP rate equal to FP rate. The curves using the known prior information of interaction are very close to the ones using the unknown prior. However, when the error rate in a dataset is extremely high, for example in contaminated data V, the ROC curve using the known prior gives more reasonable results than the one using the unknown prior. This confirmed our observation indicated in Figure 2.

### Tests on Human Data Sets

Rhodes et al. 2005 [38] collected human protein pairs from four data sources, ortholog from model organism interactome data (ortholog), genome-wide gene expression data (coexpression), protein domain data (domain), and biological functional annotation data (bio-function). Scott et al. 2007 collected more data sources in addition to the major ones in Rhodes et al. (2005) including coexpression, ortholog, domain, subcellular localication, post-translational modification co-occurrence, and protein intrinsic disorder [40]. We chose to test our approach on the data from Scott et al. (2007). The protein pairs collected from each data source are believed to be indicative of the possible interacting protein pairs, and they are measured by likelihood ratios (LRs). The protein pairs collected in each data source are firstly divided into the different feature states. The LR is then calculated for the protein pairs within that feature state by calculating the ratio of the proportion of protein pairs in the gold positive dataset to the proportion in the gold negative dataset. We chose to test on 79,441 protein pairs that have the product of LRs from all data sources greater than 100. Please review Rhodes et al. 2005 [38] and Scott et al. 2007 [40] for the principle of data collection.

We applied all the methods to integrate the scores of LRs from all the data sources of the collected human data for predicting PPIs. We tested the logistic regression model on LRs, as tested in Qi et al. (2006) [48]. We used the overlapped data with gold positive (GP) dataset and gold negative (GN) dataset to train the parameters for logistic regression model (Please review Text S1 for more information about GP and GN datasets). We predicted 39,334 PPIs using our NBEL algorithm, 16,234 PPIs using logistic regression, and 37,606 PPIs using naïve Bayes. The elucidated prior proportion of interaction for our NBEL is set as 0.5. The prior proportion of interaction was close to the empirical proportion by dividing the predictions from the naïve Bayes to the total number of protein pairs, 37,606/79,441 = 0.4734. Using a beta hyperprior can lead to an unrealistically high estimated proportion of PPIs. This is reasonable as current datasets for PPIs prediction are known to include many false positives, with rate varying from 50% to 85% [38]–[40]. As we analyzed in simulation studies, the extremely high proportion of errors in data may lead to non-informative prediction of a random probability of 0.5. An elucidated prior for the proportion of interactions however may alleviate the situation with a noticeable misclassification rate reduction.

Naïve Bayes requires a prior odds ratio, which is usually estimated by averaging the interactions per protein in the gold positive dataset. However, this value may be underestimated, since we do not know all the true interactions even in a small subset of proteins [38]–[40]. As discussed in Scott and Barton, 2007 [40], the prior odds ratio can change from 1/370 to 1/1093 across the different datasets. We picked prior odds ratio 1/400 for naïve Bayes as Scott and Barton 2007 and close to 1/381 in Rhodes et al. 2005 [38].

**Figure 5:**
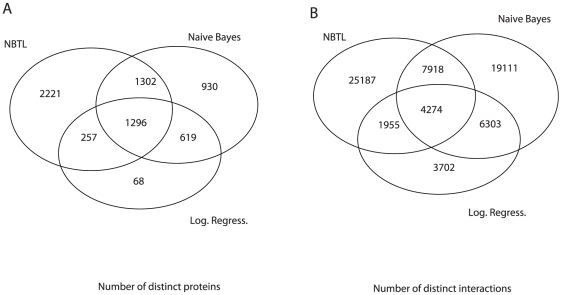
Prediction comparison among our NBEL algorithm, naïve Bayes, and logistic regression. A) listed he number of distinct and the overlapped proteins between two methods. B) listed the number of distinct and overlapped interactions among three methods.

The number of PPIs predicted from NBEL, 39,334, however, is larger than 37,606 from naïve Bayes and 16,234 from logistic regression. We further analyzed the number of distinct proteins and the distinct interactions for the identified interacting protein pairs using three methods and their overlaps, as summarized in Figure 5. It appears that most of the unique proteins and protein pairs predicted by logistic regression are also predicted by NBEL and naïve Bayes. However, we observed a more reliable performance for NBEL and logistic regression than naïve Bayes from simulation studies, we suggest being skeptical of the protein pairs that are predicted by naïve Bayes but not as much those by NBEL and logistic regression. We can also observe that many more unique proteins and protein pairs are predicted by NBEL. This may be again the result of the function of error-correction from NBEL, as discussed in detail in the Methods section. We therefore expect a more reliable prediction using NBEL than naïve Bayes. A larger number of predicted PPIs may suggest that the previous estimations may not only have a large false positive (FP) rate [1], but also may have a large false negative (FN) rate. This also suggests the necessity of considering both the FP and FN rates for PPIs predictions.

We validated the above analysis by testing on another two human PPIs datasets with high quality. Mammalian protein-protein interaction database (MIPS) [49] manually curates high-quality experimental PPI data from the scientific literature, and includes only data from individually performed experiments that are believed to have the most reliable evidence from physical interactions. We downloaded 355 human PPIs with 423 proteins from MIPS. After eliminating the protein pairs with undesignated IDs and the ones with IDs mapping problems, we had 351 protein pairs and 420 proteins. We then compared 351 protein pairs with the human data set we collected, and found 46 protein pairs that are overlapping between two datasets. Among which, we had 26 interacting proteins falling into the set that are predicted by NBEL, but had 23 by naïve Bayes and only 11 by logistic regression. Further analysis indicates that the predictions from NBEL include all the ones from naïve Bayes and logistic regression. This observation coincides to what is observed applying NBEL to our collected human dataset. Our NBEL algorithm predicted an additional portion of protein pairs that are missed by naïve Bayes and logistic regression. This indicates that the predictions using naïve Bayes and logistic regression may have a rather large number of false negatives so that a large portion of interacting protein pairs are missed and predicted as non-interacting.

We tested on another large dataset, HomoMINT [50] having 38,414 PPIs. The data together with the ones from MIPS data are summarized in Table 2, and the test results are summarized in Table 3. In Table 3, 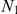 indicates the total number of protein pairs in a database that are overlapped with our collected 79,441 human PPIs; 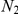 indicates the number of protein pairs in 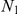 that are predicted by NBEL; 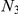 indicates the number of protein pairs in 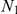 that are predicted by naïve Bayes; 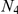 indicates the number of protein pairs in 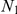 that are predicted by logistic regression. We measured true positive (TP) by calculating the proportion of protein pairs in 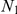 that are predicted by either naïve Bayes or NBEL. Thus, 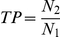 for NBEL, 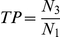 for naïve Bayes, and 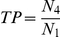 for logistic regression. False negative (FN) is simply 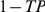.

**Table 2:** Human protein-protein interactions datasets with high quality for validating our NBEL algorithm.

Databases	Number of proteins	Number of protein pairs	Online Websites
From MIPS [49]	420	351	http://mips.helmholtz-muenchen.de/proj/ppi/
From HomoMint [50]	38,414	8,030	http://mint.bio.uniroma2.it/HomoMINT/Welcome.do
Combined dataset	38,834	8,381	

We overlapped protein pairs from each database in Table 2 with the whole collected human PPIs dataset that we tested on, and then compared the predictions out of the overlapped protein pairs for validating the performance of our NBEL algorithm.

**Table 3:** Validation by comparing NBEL algorithm with naïve Bayes via two human datasets.

		Our NBEL			Naïve Bayes			Logistic Regression		
	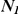	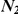	TP	FN	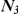	TP	FN	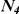	TP	FN
From MIPS [49]	46	26	56.52%	43.48%	23	50.00%	50.00%	11	23.91%	76.09%
From HomoMint [50]	1688	1235	73.16%	26.84%	1005	59.54%	40.46%	484	28.67%	71.33%

In table 3, 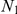 indicates the total number of protein pairs in a database that are overlapped with our collected 79,441 human PPIs; 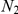 indicates the number of protein pairs in 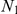 that are predicted by NBEL; 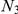 indicates the number of protein pairs in 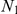 that are predicted by naïve Bayes; 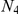 indicates the number of protein pairs in 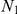 that are predicted by logistic regression.

We observe that the analysis on the second dataset has a similar pattern to that observed in the first experimental data from MIPS. The analyses from all datasets have a high true positive rate (a low false negative rate) from NBEL and a low true positive rate (a high false negative rate) from naïve Bayes and logistic regression. The overlapped predictions between three methods occupy most of the predictions from naïve Bayes and logistic regression but only a small portion from NBEL, which is consistent with the our previous analysis on our whole human data as shown in Figure 5. Again, the analysis using naïve Bayes and logistic regression missed a large portion of interacting protein pairs in having not only a large false positive rate [1] but also a large false negative rate.

## Discussion

The emergence of large-scale data has made it popular to study protein-protein interactions (PPIs) in recent years. However, one of the major issues is that a rather high proportion of false positives and negatives exist in current predictions. Data errors may occur from every data source and every stage of data collection and processing procedure. The usual approach to reduce the data errors is to minimize them from their generating source. However, such an approach can be extremely time-consuming and inefficient. Particularly, information may change as we improve our understanding in the underlying biological mechanism. A breakthrough to significantly reduce the misclassification rate is demanded for a reliable prediction of PPIs.

We proposed a nonparametric Bayes ensemble learning (NBEL) algorithm to integrate the multiple genomic data for obtaining a more powerful prediction of PPIs. Instead of the direct multiplication of scores from all data sources in naïve Bayes, our NBEL algorithm learns the distributions of interacting and non-interacting proteins within each data sources, and then automatically up-weights the informative and down-weights the less informative data sources. NBEL therefore has the function of error-correction which leads to a significant lower misclassification rate in predicting PPIs. We tested our NBEL algorithm on extensive simulations with various input data error rates varying from 0% to >70%, which mimic a rather high false positive rate >70% that is reported in previous PPIs predictions. Our simulation results indicated that our NBEL algorithm has a much lower misclassification rate, with the rate reduction varying from 7% to 25% from naïve Bayes and logistic regression. This suggests that NBEL is significantly more robust than naïve Bayes and logistic regression to highly contaminated data. Such a function becomes stronger as the number of data sources increases. Our tests on a large human data set indicate that NBEL predicts a larger number of PPIs than naïve Bayes and logistic regression, which are validated using two reliable experimental PPIs data. This indicates that rather high not only FP rate but also FN rate may exist in previous studies. This also suggests the importance of evaluating both the FP and FN rates in PPIs prediction.

We successfully demonstrated the feasibility of predicting high-throughput PPIs computationally, with substantially reduced false positives and false negatives. Our work may inspire people to utilize computational approaches to correct data errors for any problem in the field of computational biology that needs predictions from multiple data sources. The ability of predicting large numbers of PPIs both reliably and automatically may speed up PPIs prediction. Such a reliable prediction may provide a solid platform to other related studies. One example is the study of protein functions prediction since the group of protein pairs that tend to interact with each other may have similar functions. Another example is the study of roles of PPIs in disease susceptibility as the dynamic changes of PPIs may relate to disease causality.

There are still future works left for obtaining more complete and reliable inferences of PPIs. Current estimates of PPIs have a very low coverage [1]. The set of known interactions is even less representative of the whole network since the subset of interactions is by no means random. The analysis also showed that there is little overlap between the high-throughput datasets [1]. Paradoxically, some attempts to increase data quality, for example, multiple validations, make these biases more severe [1]. Although Lu et al. 2005 [37] indicated no appreciable dependence between any possible pairs of data sources for yeast. Information sharing does exist in the different levels among data sources for human. For example, it is believed that the interacting protein pairs sharing the same biological process may also have physical associations between the enriched domains. Redundant information thus exists among the data sources of the biological functional annotation data and the protein domain data [38]. This invalidates our assumption of conditional independence in the different data sources given the unknown PPI status. Although some manual procedure as a semi-naïve Bayes is proposed in current works [22], [35]–[38], [40]–[41] to reduce such dependency, dependency exists more or less among any two of the disparate data sources. An effective integration method releasing the restriction of the conditional dependence is therefore demanded. Furthermore, since the network of PPIs is essentially time-evolving, an approach that is able to model the PPIs dynamically is desirable.

## Methods

In this section, we describe our NBEL method to integrate the likelihood ratios (scores) from the disparate data sources for the prediction of PPIs.

Let **Y** denote an 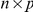 matrix, with rows corresponding to different protein pairs and columns to different types of scores from different data sources, with high values of the scores providing evidence of an interaction between the proteins. Typical analyses of protein interaction networks are based on one type of data, but here we propose a nonparametric Bayes latent class discriminant analysis approach for combining information from different data sources. We refer to this as ensemble learning following terminology in the machine learning literature. Let 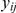 denote the score in row *i* and column *j* of matrix **Y** and let 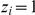 if the 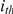 pair interacts with 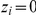 otherwise.

Our nonparametric Bayes ensemble learning (NBEL) model assumes that
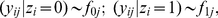
(1)where 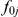 is the unknown distribution of the 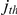 score across protein pairs that do not interact, and 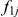 is the unknown distribution of the 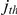 score across protein pairs that do interact, for 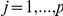. For identifiability, we assume that 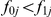, denoting that 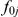 is stochastically less than 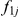. Following a Bayesian approach, we place priors on the unknown distributions 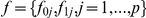.

In particular, we characterize each distribution using an infinite mixture model with
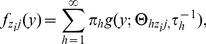
(2)where g(.) is a parametric kernel (e.g., Gaussian), 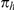 is a mixture weight on component *h*, 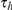 is a precision parameter specific to mixture component *h*, and 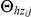 are location parameters specific to mixture component *h*, interaction status *z*, and score type *j*. It is well known that mixtures are extremely flexible. By allowing the kernel locations for each component to vary flexibly with interaction status and score type, we obtain a highly flexible model. The stochastic ordering restriction can be enforced by restricting 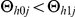 for all *h*, *j*.

Dunson and Peddada 2008 [51] propose a restricted dependent Dirichlet process (rDDP) prior for modeling of unkown stochastically ordered distributions of the form shown in (2). However, they do not consider the case in which the stochastic ordering is over latent groups or cases in which data are available from different data sources. Conditionally on the data **Y** and the distributions *f*, the posterior probability of an interaction in pair *i* is
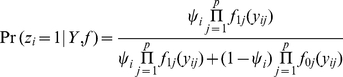
(3)where 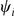 is the prior probability of an interaction in pair *i*. This prior probability can be set to 0.5 to be uninformative, or one can incorporate available information outside of that included in the score 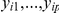 in the choice of 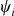. Expression (3) describes that the information, such as the sharing and dependence among protein pairs, is borrowed via the normal mixture model and integrated for predicting protein-protein integrations.

The information can be transferred across the different protein pairs within columns (data sources). The distributions for interacting protein pairs and non-interacting protein pairs are learnt via the normal mixture model in expression (2). If only one data source were available (*p* = 1), there would be no ability to predict the interaction status latent variables 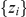 and separately estimate the interacting and non-interacting score distributions without labeled data in which 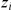 was known without error for a training subset. However, when repeated scores are available (*p*>1), we obtain identifiability through the dependence structure in the multiple scores. In particular, the model will automatically interpret multiple scores that are high as evidence that the pair is more likely to be interacting. Essentially, the shared dependence on the latent class 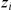 induces dependence in the multiple scores 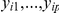, allowing us to nonparametrically identify the different score densities under the stochastic ordering restriction. If a particular score (say score *j* = 3) tends to be unreliable, then it will have relatively low correlation with the other scores marginalizing out the latent 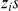, and hence the separation between 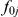 and 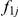 will be small. This small separation and low correlation will automatically lead to unreliable data sources being down-weighted and potentially even effectively excluded. This type of flexible adaptive weighting should substantially improve misclassification rates, and hence reduce false positives. This will be assessed through simulation studies in Section Results.

To complete a Bayesian specification of the model, we choose 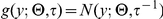, the univariate Gaussian distribution centered on 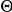 with precision 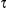. In addition, following an rDDP specification (Dunson and Peddada 2008 [51]), we let
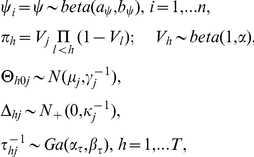
where 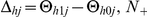 denotes a normal distribution truncazted below by zero, and 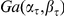 denotes the gamma distribution. Letting 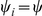 for simplicity, 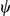 represents the prior probability that a random selected protein pair is interacting. By choosing a beta hyper-prior on 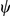, we let the data inform about the proportion of interacting pairs. Normalizing the scores prior to analysis within each column of Y, we recommend the following default hyperparameter values, 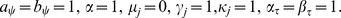


We propose a blocked Gibbs sampler to estimate the posterior probabilities of unknowns (Ishwaran and James 2001 [52]) (Please find the details from Text S1). Our focus is on inference on the protein interactions based on the marginal posterior probabilities of 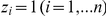, which can be calculated using a Rao-Blackwellized approach. In particular, discarding a burn-in to allow convergence, we average the conditional posterior probabilities 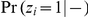 for each *i* across a large number of MCMC iterations. Under 0–1 loss, the Bayes optimal classification rule sets 
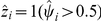
 where 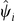 is the estimated posterior probability of 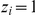. We recommend collecting 5,000 iterations, with the first 1,000 iterations discarded as a default.

## Supporting Information

Text S1 **The text file includes the parameters used to generate the simulated datasets, posterior computation, and the description of Gold Standard datasets.**
(PDF)
