# Supplementary material for: Bayesian Inference for Genomic Data Integration Reduces Misclassification Rate in Predicting Protein-Protein Interactions
Source: PLoS Comput Biol. 2011 Jul 28;7(7):e1002110. doi: 10.1371/journal.pcbi.1002110 (PMC3145649; doi:10.1371/journal.pcbi.1002110)
Supplement: Text S1 — The text file includes the parameters used to generate the simulated datasets, posterior computation, and the description of Gold Standard datasets. (PDF) [file pcbi.1002110.s001.pdf]

Supplementary Material for *Bayesian Inference for Genomic Data Integration Reduces Misclassification Rate in Prediction of Protein-Protein Interactions*

Chuanhua Xing[1] and David B. Dunson[2]
[1]Department of Biostatistics and Bioinformatics, Duke University, Durham, NC.
[2]Department of Statistics, Duke University, Durham, NC.

## 1 Parameters Used to Generate Data

Equation (2) in Methods section used to generated simulated data is copied here again,

$$f_{z_j}(y) = \sum_{h=1}^{\infty} \pi_h g(y; \Theta_{hzj}, \tau_h), \quad (1)$$

where $g(\cdot)$ is a parametric kernel (e.g., Gaussian), $\pi_h$ is a mixture weight on component $h$, $\tau_h$ is a precision parameter specific to mixture component $h$, and $\Theta_{hzj}$ are location parameters specific to mixture component $h$, interaction status $z$, and score type $j$. The parameters are listed in the following table.

Table 1: The parameters used to generate the simulated datasets. The mathematical expression is given by Equation (2) in Materials and Methods section.

| Data Sources | | h=1 | | h=2 | | h=3 | |
|---|---|---|---|---|---|---|---|
| | | $\pi_1$ | $(\Theta, \tau^{-1})$ | $\pi_2$ | $(\Theta, \tau^{-1})$ | $\pi_3$ | $(\Theta, \tau^{-1})$ |
| j=1 | z=1 | 0.2 | (5,1) | 0.3 | (7,2.25) | 0.5 | (10,4) |
| | z=0 | 0.2 | (2,16) | 0.3 | (4,9) | 0.5 | (4,4) |
| j=2 | z=1 | 0.1 | (4,0.25) | 0.3 | (6,1) | 0.6 | (7,2.25) |
| | z=0 | 0.1 | (3,9) | 0.3 | (4,2.25) | 0.6 | (2,1) |
| j=3 | z=1 | 0.1 | (60,1) | 0.2 | (65,9) | 0.7 | (70,25) |
| | z=0 | 0.1 | (55,100) | 0.2 | (50,25) | 0.7 | (58,9) |
| j=4 | z=1 | 0.2 | (10,1) | 0.6 | (15,4) | 0.2 | (20,9) |
| | z=0 | 0.2 | (8,49) | 0.6 | (5,16) | 0.2 | (7,4) |

## 2 Posterior Computation

We propose a blocked Gibbs sampler for posterior computation [1]. This approach can be used for estimation of the posterior probability of $z_i = 1$ for $i = 1, \ldots, n$, which provides an easy-to-interpret weight of evidence of an interaction between proteins, fully accommodating uncertainty in the model specification and borrowing information across different data. The blocked Gibbs sampler iterates between the following steps after choosing initial values,

1. Update $z_i$ by sampling from the Bernoulli full conditional posterior distribution, $\Pr(z_i = 1 \mid -)$, for $i = 1, \ldots, n$,

$$\frac{\psi \prod_{j=1}^{p} \mathrm{N}(y_{ij}; \Theta_{S_{ij}1j}, \tau_{S_{ij}j}^{-1})}{\psi \prod_{j=1}^{p} \mathrm{N}(y_{ij}; \Theta_{S_{ij}1j}, \tau_{S_{ij}j}^{-1}) + (1-\psi) \prod_{j=1}^{p} \mathrm{N}(y_{ij}; \Theta_{S_{ij}0j}, \tau_{S_{ij}j}^{-1})}.$$

2. Update $\psi$ from a beta conditional posterior distribution,

$$\mathrm{Beta}\bigg(1 + \sum_{i=1}^{n} z_i, 1 + \sum_{i=1}^{n}(1 - z_i)\bigg).$$

3. Update $S_{ij} \in \{1, \ldots, T\}$, the allocation of observation $y_{ij}$ to mixture component $\{1, \ldots, T\}$, from a multinomial conditional posterior distribution. Here, the infinite summation in Equation (1) is truncated to the first $T$ terms by letting $V_T = 1$, following the theoretical justification of [1].

$$\Pr(S_{ij} = h \mid -) = \frac{\pi_h \mathrm{N}(y_{ij}; \Theta_{hz_ij}, \tau_{hj}^{-1})}{\sum_{l=1}^{T} \pi_l \mathrm{N}(y_{ij}; \Theta_{lz_ij}, \tau_{lj}^{-1})}, \quad h = 1, \ldots, T.$$

4. Update $V_h$ for $h = 1, \ldots, T-1$ from a beta full conditional posterior distribution,

$$\mathrm{Beta}\bigg(1 + \sum_{i=1}^{n}\sum_{j=1}^{p} 1(S_{ij} = h), \alpha + \sum_{i=1}^{n}\sum_{j=1}^{p} 1(S_{ij} > h)\bigg).$$

5. Update $\Theta_{h0j}$, for $h = 1, \ldots, T$ and $j = 1, \ldots, p$, from a normal conditional posterior $\mathrm{N}(E_{h0j}, V_{h0j})$, with

$$\begin{aligned} E_{h0j} &= V_{h0j}\bigg\{\mu_j\gamma_j + \tau_{hj}\sum_{i=1}^{n} 1(S_{ij} = h)(y_{ij} - z_i\Delta_{hj})\bigg\}, \\ V_{h0j} &= \bigg\{\gamma_j + \tau_{hj}\sum_{i=1}^{n} 1(S_{ij} = h)\bigg\}^{-1}. \end{aligned}$$

6. Update $\Delta_{hj}$, for $h = 1, \ldots, T$ and $j = 1, \ldots, p$, from $\mathrm{N}_+(E_{h1j},\ V_{h1j})$ with

$$\begin{aligned} E_{h1j} &= V_{h1j}\bigg\{\tau_{hj}\sum_{i=1}^{n} 1(S_{ij} = h) z_i (y_{ij} - \Theta_{h0j})\bigg\}, \\ V_{h1j} &= \bigg\{\kappa_j + \tau_{hj}\sum_{i=1}^{n} 1(S_{ij} = h) z_i\bigg\}^{-1}. \end{aligned}$$

7. Update $\tau_{hj}$, for $h = 1, \ldots, T$ and $j = 1, \ldots, p$, from

$$\mathrm{Ga}\bigg(a_\tau + \frac{1}{2}\sum_{i=1}^{n} 1(S_{ij} = 1), b_\tau + \frac{1}{2}\sum_{i:S_{ij}=1} (y_{ij} - \Theta_{h1j})^2\bigg).$$

Each of these steps involves sampling from standard distributions, and hence the implementation is quite simple and efficient. The samples converge to a stationary distribution that is the joint posterior distribution of the unknowns. Our focus is on inference on the protein interactions based on the marginal posterior probabilities of $z_i = 1$, which can be calculated using a Rao-Blackwellized approach. In particular, discarding a burn-in to allow convergence, we average the conditional posterior probabilities in step 1 for each $i$ across a large number of MCMC iterations. Under 0-1 loss, the Bayes optimal classification rule sets $\widehat{z}_i = 1(\widehat{\psi}_i > 0.5)$, where $\widehat{\psi}_i$ is the estimated posterior probability of $z_i = 1$. We recommend collecting 5,000 iterations, with the first 1,000 iterations discarded as a default.

# 3 Gold Standard Datasets

We obtained the gold positive (GP) dataset by downloading the data from Human Protein Reference Dataset (HPRD) (http://www.hprd.org) (Prasad et al. 2009 [2]; Mishra et al. 2006 [3]; Peri et al. 2003 [4]). We downloaded 37107 protein protein interaction pairs with 9463 proteins from HPRD in Release 7, June 29, 2010. We had 8328 proteins and 31864 interaction pairs left after removing the duplicate and self-interactions, and they therefore composed our gold positive (GN) dataset. We composed the gold standard negative dataset by downloading the data from Gene Ontology Consortium (http://www.geneontology.org) (The Gene Ontology Consortium 2000 [5]; Harris et al. 2004 [6]). For all the protein pairs, one protein was assigned to the plasma membrane cellular component (749 proteins), and the other was assigned to the nucleus cellular component (1054 proteins). A few proteins that were assigned in both were removed, although we expect the proteins assigned to the plasma membrane cellular component can seldom interact with the ones assigned to the nucleus cellular component.

# References


[1] Ishwaran H and James LF (2001) Gibbs sampling methods for stick-breaking priors. J Am Statist Assoc., 96:16173.

[2] Prasad TSK, Goel R, Kandasamy K, Keerthikumar S, Kumar S, Mathivanan S, Telikicherla D, Raju R, Shafreen B, Venugopal A, et al. (2009). Human Protein Reference Database - 2009 update. Nucleic Acids Res. 37: D767-D772.

[3] Mishra G, Suresh M, Kumaran K, Kannabiran N, Suresh S, Bala P, Shivkumar K, Anuradha N, Reddy R, Raghavan TM (2006). Human Protein Reference Database - 2006 Update. Nucleic Acids Res. 34: D411-D414.

[4] Peri S, Navarro JD, Amanchy R, Kristiansen TZ, Jonnalagadda CK,Surendranath V, Niranjan V, Muthusamy B, Gandhi TK, Gronborg M, et al. (2003). Development of human protein reference database as an initial platform for approaching systems biology in humans. Genome Res 13: 2363-2371.

[5] The Gene Ontology Consortium. (2000). Gene Ontology: tool for the unification of biology. Nature Genet. 25: 25-29.

[6] Harris MA, Clark J, Ireland A, Lomax J, Ashburner M, Foulger R, Eilbeck K, Lewis S, Marshall B, Mungall C et al. (2004). The Gene Ontology (GO) database and informatics resource. Nucleic Acids Res 32: D258-D261.
